# Supplementary material for: The impact of social deprivation on development and progression of diabetic kidney disease
Source: HRB Open Res. 2024 Aug 8;7:53. [Version 1] doi: 10.12688/hrbopenres.13941.1 (PMC11411243; doi:10.12688/hrbopenres.13941.1)
Supplement: Peer review history

# Reviewer report: Reviewer response for version 1

**DOI:** 10.21956/hrbopenres.15297.r42243

**Published:** 2024-09-18

**Authors:** Christian Stevns Hansen

**Affiliations:** 1 Steno Diabetes Center Copenhagen, Herlev, Denmark

Review of  HRB Open Research: Study protocol: The impact of social deprivation on development and progression of diabetic kidney disease [version 1; peer review:1 approved]

**Overall considerations**

 The protocol describes an important topic: social deprivation indices and diabetic kidney disease. If these links are established in the Irish population new focused interventions are warranted in the deprived areas. The protocol is clear and concise.  

**Abstract**

 Clear and concise

**Introduction**

 Good background overview and clear and valid objectives

**Study design**

 Is appropriate. It seems that the study database is appropriate in size, follow-up time and detail to allow for the analyses to be used.

**Statistical analyses**

 Are appropriate.

**Results**

 Is appropriately planned.

**Conclusion**

 Is appropriately planned.

Is the study design appropriate for the research question?

Yes

Is the rationale for, and objectives of, the study clearly described?

Yes

Are sufficient details of the methods provided to allow replication by others?

Yes

Are the datasets clearly presented in a useable and accessible format?

Yes

Reviewer Expertise:

Diabetes epidemiology. Diabetic neuropathy. randomized trails. Risk factor analyses

I confirm that I have read this submission and believe that I have an appropriate level of expertise to confirm that it is of an acceptable scientific standard.

# Reviewer report: Reviewer response for version 1

**DOI:** 10.21956/hrbopenres.15297.r41919

**Published:** 2024-09-10

**Authors:** Salil Deo

**Affiliations:** 1 Case School of Medicine, Case Western Reserve University, Cleveland, Ohio, USA

This is a retrospective study that will use the DIAMOND data to evaluate the association between social determinants and kidney function decline in Galway Ireland. The study introduction is well written. CKD is an important adverse event among people with T2D. Understanding the longitudinal decline in kidney function would be important to understand in this cohort & the importance of SDoH in this regard is also important. 

 I congratulate the authors on this well written study protocol & look forward to the results.

Is the study design appropriate for the research question?

Yes

Is the rationale for, and objectives of, the study clearly described?

Yes

Are sufficient details of the methods provided to allow replication by others?

Yes

Are the datasets clearly presented in a useable and accessible format?

Yes

Reviewer Expertise:

Cardiovascular disease, Social determinants of health, Cardiac Surgery, Risk prediction models

I confirm that I have read this submission and believe that I have an appropriate level of expertise to confirm that it is of an acceptable scientific standard.
